# Supplementary material for: One Year after Mild COVID-19: The Majority of Patients Maintain Specific Immunity, But One in Four Still Suffer from Long-Term Symptoms
Source: J Clin Med. 2021 Jul 27;10(15):3305. doi: 10.3390/jcm10153305 (PMC8347559; doi:10.3390/jcm10153305)
Supplement: Supplementary file 1 [file jcm-10-03305-s001.zip › jcm-1297310-supplementary.pdf]

Table S1: Frequency of persistent symptoms after mild COVID-19 (total number: 83).

	>1 month	>3 months	>6 months	>12 months
**Any persistent symptom**	51 (61%)	41 (49%)	36 (43%)	23 (28%)
Loss of smell	24 (29%)	21 (25%)	16 (19%)	9 (11%)
Loss of taste	18 (22%)	16 (19%)	14 (17%)	8 (10%)
Concentration disorder	16 (19%)	15 (18%)	14 (17%)	10 (12%)
Headache	15 (18%)	15 (18%)	15 (18%)	11 (13%)
Dyspnoe (excertion)	15 (18%)	10 (12%)	10 (12%)	6 (7%)
Back pain	9 (11%)	9 (11%)	9 (11%)	4 (5%)
Muscle pain	7 (8%)	7 (8%)	7 (8%)	5 (6%)
Thoracic pain	5 (6%)	3 (3%)	3 (3%)	0 (0%)

Table S2. Correlation analysis between humoral and cellular immunity of non-vaccinated participants at 12 months follow-up. THC: T helper cells. Nab: neutralizing antibodies, SI: stimulation index.

	**INFg ELISPOT assay (SI)**			**Il-2 ELISPOT assay (SI)**			**AIM assay (THC%)**		
	R^2	cc	p value	R^2	cc	p value	R^2	cc	p value
**IgA assay (value)**	0.001	0.136	0.242	0.003	-0.053	0.664	0.005	0.207	0.086
**IgG assay (value)**	0.151	0.355	**0.002**	0.061	0.256	**0.032**	0.050	0.264	**0.027**
**Nab assay (titer)**	0.175	0.392	**>0.001**	0.062	0.278	**0.020**	0.075	0.297	**0.013**

UNIVERSITÄTSKLINIKUM
AUGSBURG

COVID-19 Questionnaire

Name/Date of birth: Date:

	No	Yes	Description, if you answered Yes
Have you had any of the following since recovering from COVID-19: episodes of fever?			When? How long? Completely recovered?
any influenza or influenza-like infection?			
After acute COVID-19, did you suffer for a longer time period / until now from… Loss of the sense of smell ?			How long? Completely recovered?
Loss of the sense of taste ?			
Difficulty in concentrating ?			
Headache ?			
Back pain ?			
Muscle pain ?			
Shortness of breath ?			At rest? During exercise?
Do you suffer from any new illness or discomfort since recovering from COVID-19 ?			
Were thrombosis or lung embolism diagnosed at any time during or after COVID-19 ?			
Did you take blood-thinning medication during COVID-19 (Aspirin, Heparin, or others) ?			
Do you take any new medication since recovering from COVID-19 ?			

Please mark on a scale of - 5 to 0 points how your resilience and fitness has developed since the COVID-19 disease until now. A score of - 5 means that you still feel as ill as you did during the COVID-19 period, and 0 means that you have regained the full strength that you had before COVID-19.

- 5	- 4	- 3	- 2	- 1	0	
Still sick					*completely recovered*	*even fitter than before*

Thank you for your help!

Universitätsklinikum Augsburg A.ö.R, Standort: Stenglinstraße 2, 86156 Augsburg | Sauerbruchstraße 6, 86179 Augsburg, www.uk-augsburg.de
Vorstand: Vorstandsvorsitzender & Ärztlicher Direktor: Prof. Dr. med. Dr. h.c. Michael Beyer,
Kaufmännischer Direktor: Michael Bungarten, Pflegedirektorin: Susanne Arnold, Gründungsdekanin: Prof. Dr. med. Martina Kadmon
USt-ID: DE 320 913 762; Stadtsparkasse Augsburg: IBAN DE92 7205 0000 0000 0680 80 / BIC AUGSDE77XXX;
Kreissparkasse Augsburg: IBAN DE11 7205 0101 0380 0032 10 / BIC BYLADEM1AUG; Deutsche Postbank: IBAN DE67 7601 0085 0118 9368 59 / BIC PBNKDEFFXXX

Figure S1. Structured questionnaire for follow-up after COVID-19.
